# Dual 2-Hydroxypropyl-β-Cyclodextrin and 5,10,15,20-Tetrakis (4-Hydroxyphenyl) Porphyrin System as a Novel Chiral-Achiral Selector Complex for Enantioseparation of Aminoalkanol Derivatives with Anticancer Activity in Capillary Electrophoresis

**DOI:** 10.3390/molecules26040993

**Published:** 2021-02-13

**Authors:** Błażej Grodner, Mariola Napiórkowska

**Affiliations:** 1 Chair and Department of Biochemistry and Pharmacogenomics, Medical University of Warsaw, 1 Banacha Str., 02-097 Warsaw, Poland; 2 Chair and Department of Biochemistry, Medical University of Warsaw, 1 Banacha Str., 02-097 Warsaw, Poland; mariola.napiorkowska@wum.edu.pl

**Keywords:** anticancer drug, capillary electrophoresis, chiral selectors, drug monitoring

## Abstract

In this study, a complex consisting of 2-hydroxypropyl-β-cyclodextrin and 5,10,15,20-tetrakis (4-hydroxyphenyl) porphyrin, (named dual chiral-achiral selector complex) was used for the determination of two novel potential anticancer agents of (I) and (II) aminoalkanol derivatives. This work aimed at developing an effective method that can be utilized for the determination of I (S), I (R), and II (S) and II (R) enantiomers of (I) and (II) compounds through the use of a dual chiral-achiral selector complex consisting of hydroxypropyl-β-cyclodextrin and 5,10,15,20-tetrakis (4-hydroxyphenyl) porphyrin system by applying capillary electrophoresis. This combination proved to be beneficial in achieving high separation selectivity due to the combined effects of different modes of chiral discrimination. The enantiomers of (I) and (II) compounds were separated within a very short time of 3.6–7.2 min, in pH 2.5 phosphate buffer containing 2-hydroxypropyl-β-cyclodextrin and 5,10,15,20-tetrakis (4-hydroxyphenyl) porphyrin system at a concentration of 5 and 10 mM, respectively, at 25 °C and +10 kV. The detection wavelength of the detector was set at 200 nm. The LOD for I (S), I (R), II (S), and II (R) was 65.2, 65.6, 65.1, and 65.7 ng/mL, respectively. LOQ for I (S), I (R), II (S), and II (R) was 216.5, 217.8, 217.1, and 218.1 ng/mL, respectively. Recovery was 94.9–99.9%. The repeatability and reproducibility of the method based on the values of the migration time, and the area under the peak was 0.3–2.9% RSD. The stability of the method was determined at 0.1–4.9% RSD. The developed method was used in the pilot studies for determining the enantiomers I (S), I (R), II (S), and II (R) in the blood serum.

## 1. Introduction

Separation of chiral compounds is very important in medical and pharmaceutical research as it allows studying the content of individual enantiomers in drugs and their concentrations in the human body and thus to determine the pharmacokinetic and pharmacodynamic parameters. Chiral compounds play a major role in drug–receptor interactions, in which one enantiomer of a racemic pair confers the desired pharmacological activity, while the other may exhibit negative side effects in the worst case [1,2,3]. In chiral compounds, numerous drugs, which have one or multiple asymmetric centers, occur as an enantiomer pair [4,5,6,7,8,9,10]. Therefore, it is necessary for the researchers to have knowledge of the composition of enantiomeric medicinal substances [11]. The most popular technique used for the determination of biological substances is the high-performance liquid chromatography. However, capillary electrophoresis (CE) is becoming an appealing alternative as it possesses a number of benefits such as high-speed analysis, low sample consumption, and high separation efficiency. CE has already been demonstrated to have high potential in chiral separations [12], yielding very good results in the analyses of various pharmaceutically active substances. As we know, interactions occurring between chiral selectors and an enantiomer are the basis for separation in CE. For achieving effective chiral separation in CE, the chiral selector should be added to the run buffer. Among the great variety of compounds that can be used as a chiral selector in CE are cyclodextrins (CDs), antibiotics, crown ethers, cyclofructans, and polysaccharides. CDs are one of the most commonly used chiral selectors [13,14,15]. Antibiotics [16,17,18,19,20,21], CD derivatives [22,23,24,25,26,27], chiral ligand exchangers [28,29,30,31,32,33,34], crown ethers [35,36,37,38], polysaccharides [39,40,41,42], and proteins [43,44,45] have also been employed depending on the properties of the enantiomers to be separated.

**Figure 1:**
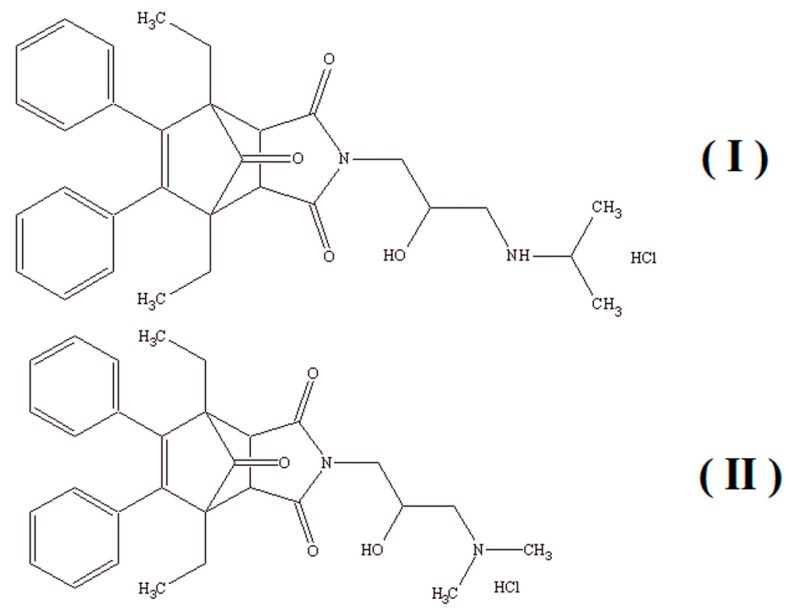
Chemical structures of 4-[2-hydroxy-3-(propan-2-ylamino)propyl]-1,7-diethyl-8,9-diphenyl-4-azatricyclo-[5.2.1.0^2,6^]dec-8-ene-3,5,10-trione hydrochloride (**I**) and 4-[3-(dimethylamino)-2-hydroxypropyl]-1,7-diethyl-8,9-diphenyl-4-azatricyclo[5.2.1.0^2,6^]dec-8-ene-3,5,10-trione hydrochloride (**II**).

This study aimed to develop a new CE method characterized by high sensitivity and having the potential for rapid separation of the enantiomers I(R), I(S), and II(R), II(S) of the patented 4-[2-hydroxy-3-(propan-2-ylamino)propyl]-1,7-diethyl-8,9-diphenyl-4-azatricyclo-[5.2.1.02,6]dec-8-ene-3,5,10-trione hydrochloride (I) and 4-[3-(dimethylamino)-2-hydroxypropyl]-1,7-diethyl-8,9-diphenyl-4-azatricyclo[5.2.1.02,6]dec-8-ene-3,5,10-trione hydrochloride (II) (Figure 1) exhibiting anticancer activity [46].

**Figure 2:**
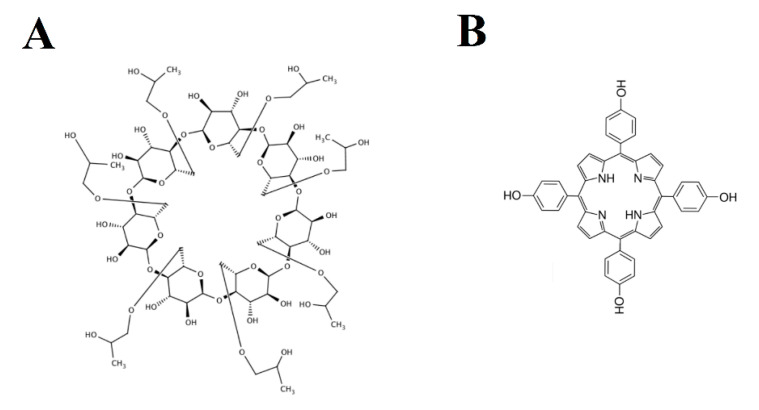
Chemical structures of 2-hydroxypropyl-β-cyclodextrin (**A**), 5,10,15,20-tetrakis (4-hydroxyphenyl) porphyrin (**B**).

These compounds came from research into the cytotoxicity and anticancer properties notificated in a patent in 2012 [46]. Determination of compounds (I) and (II) using a CD derivative as a chiral selector has already been described in the previous work [47]. In the present study, we used the dual 2-hydroxypropyl-β-cyclodextrin and 5,10,15,20-tetrakis (4-hydroxyphenyl) porphyrin (Figure 2) chiral-achiral selector complex to investigate the combined effect of these two types of selectors on the enantioseparation of the model compounds. The developed method was found to be very effective and was used in the pilot studies for the determination of enantiomers in the blood serum.

## 2. Materials and Methods

### 2.1. Chemicals and Reagents

The synthesis of the compounds as well as their spectroscopic analysis (nuclear magnetic resonance and mass spectrometry) has been explained in patent application [46]. The purity of reference compounds was greater than 98%, while other analytes used in separations had 99% purity [47]. Phosphate buffer used in the study was obtained from Beckman-Coulter (Brea, USA), while the other chemicals were purchased from Sigma-Aldrich: γ-, α-, β-, and 2-hydroxypropyl-β-cyclodextrin (St. Louis, USA); deionized water and 5,10,15,20-tetrakis (4-hydroxyphenyl) porphyrin (Darmstadt, Germany); and ethyl acetate and hexane (Steinheim, Germany). The serum samples were obtained from 10 healthy drug-free volunteers.

### 2.2. Instrumentation

A Beckman Coulter P/ACE MDQ CE system, which contained an autosampler as well as a UV/Visible detector, was used for the separation. Karat software version 32 was used for controlling the CE parameters [47]. An eCAP fused-silica capillary (total length: 30 cm, effective length: 20 cm, ID: 50 μm, OD: 375 μm) was employed.

### 2.3. Sample Preparation

Stock solutions for the **I** (*S*), **I** (*R*), and **II** (*S*) and **II** (*R*) enantiomers, at the concentration of 10,000 ng/mL were prepared by dissolving of these substances in methanol. Then, from these solutions, calibration solutions with specific concentrations were prepared. These solutions were stored at −15°C. The analytes were found to be stable in the solution for around 30 days in the previous studies. Samples for the preparation of the working standards and the quality controls were prepared from the serum obtained from 10 healthy drug-free volunteers. The calibration solutions of 50, 100, 500, 1000, 5000, and 10,000 ng/mL were prepared in water and in serum, by adding appropriate amounts of the analyte stock solutions to the blank serum samples. Serum from healthy volunteers was a medium for dilution of the compounds and did not contain the tested compounds. As described previously [47], extraction was carried out with 500 μL of serum, 100 μL of **I** (*S*) and **I** (*R*), 100 μL of **II** (*S*) and **II** (*R*), and 5 mL of the *n*-hexane–ethyl acetate mixture (90:10, % *v*/*v*). These solutions were mixed and shaken well for around 4 min. After centrifugation (at 5 min, 1000× *g*), the organic layer was removed and subjected to evaporation under a nitrogen stream at 37 °C. Then, the obtained sample was mixed with deionized water (100 μL) and introduced on the capillary [47].

### 2.4. CE Conditions

Electrophoretic separations were carried out in the eCAP fused-silica capillary (total length: 30 cm, effective length: 20 cm, ID: 50 μm, OD: 375 μm) [47]. The enantiomers **I** (*S*), **I** (*R*), **II** (*S*), and **II** (*R*) were analyzed with sodium dihydrogen phosphate (25 mM), which contained 2-hydroxypropyl-β-cyclodextrin and 5,10,15,20-tetrakis (4-hydroxyphenyl) porphyrin system at a concentration of 5 and 10 mM, respectively, as background electrolyte (BGE); the system was adjusted to a pH of 2.5 by adding phosphoric acid. The parameters of the detector were set as follows: Wavelength: 200 nm, temperature: 25 °C, and voltage: +10 kV.

New capillaries were flushed with 1 M NaOH (15 min, 10 psi), water (15 min, 10 psi), and BGE (20 min, 10 psi). Finally, the system was electroconditioned with the running buffer by applying the separation voltage (+10 kV) for 20 min. Between workdays, the capillary was conditioned by rinsing successively with 0.1 M NaOH (5 min, 10 psi), water (5 min, 10 psi), and BGE (10 min, 10 psi). Samples were hydrodynamically injected at a pressure of 2 psi for 6 s. A separation voltage of 10 kV (normal polarity anode at the injection capillary end) was applied for performing the electrophoretic separations at 25°C. Between runs, the capillary was rinsed for 2 min with water and for 3 min with BGE. At the end of every analysis, the working BGE was refreshed to ensure optimum separation repeatability. After extraction with n-hexane–ethyl acetate mixture (90:10, % *v*/*v*) and dissolving in 0.1 mL of aqueous solution, the serum samples were injected automatically using pressure (2 psi on sample solution for 5 s). The vials having both cathode and anode buffers were emptied late in the day, and the running buffer was refilled at the start of the following working day, before analysis, as indicated by the earlier described procedure [47].

## 3. Results and Discussion

### 3.1. Optimization

**Figure 3:**
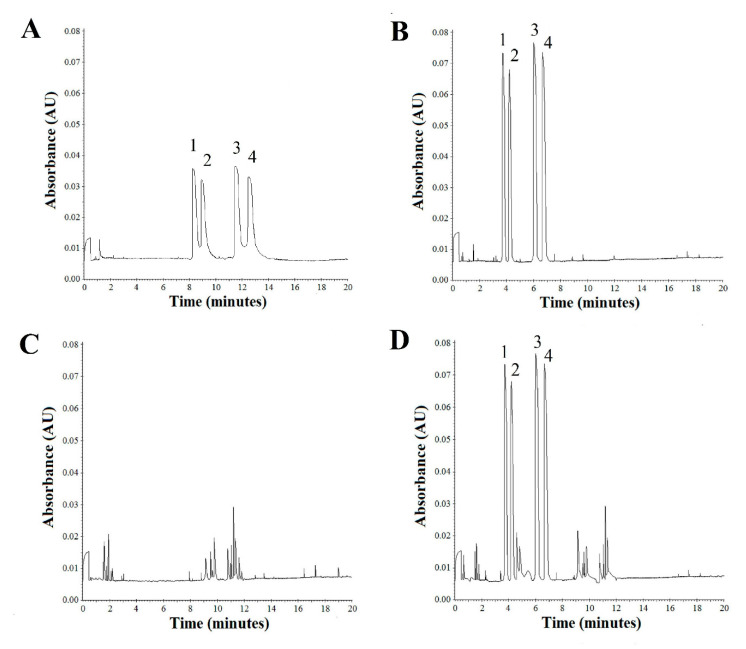
Separation of the (1) **I** (*S*), (2) **I** (*R*), (3) **II** (*S*), and (4) **II** (*R*) enantiomers in the 25 mM phosphate buffer of pH 2.5 containing 2-hydroxypropyl-β-cyclodextrin (**A**), 2-hydroxypropyl-β-cyclodextrin and 5,10,15,20-tetrakis (4-hydroxyphenyl) porphyrin system at the concentration of 5 and 10 mM, respectively (**B**), blank serum (**C**), and serum with addition of **I** (*S*), **I** (*R*) and **II** (*S*), **II** (*R*) enantiomers of the aminoalkanol derivatives (**D**).

In this study, three kinds of CDs, namely 2-hydroxypropyl-α-cyclodextrin, 2-hydroxypropyl-β-cyclodextrin, and 2-hydroxypropyl-γ-cyclodextrin, and one porphyrin derivative (5,10,15,20-tetrakis (4-hydroxyphenyl) porphyrin) were screened at different pH values as potential chiral selector candidates, either alone or in combinations. Among the tested CDs, only 2-hydroxypropyl-β-cyclodextrin allowed a baseline separation of the enantiomers of compounds (**I**) and (**II**). The best results were achieved when 2-hydroxypropyl-β-cyclodextrin was used at a concentration of 5 mM (Figure 3A). Other phosphate buffers, containing 2-hydroxypropyl-α-cyclodextrin and 2-hydroxypropyl-γ-cyclodextrin at a concentration of 5 mM, did not allow satisfactory separation of compounds (**I**) and (**II**) into individual enantiomers. Therefore, for the initial separation of the enantiomers **I** (*S*), **I** (*R*), **II** (*S*), and **II** (*R*), 2-hydroxypropyl-β-cyclodextrin was chosen as the first chiral selector.

**Figure 4:**
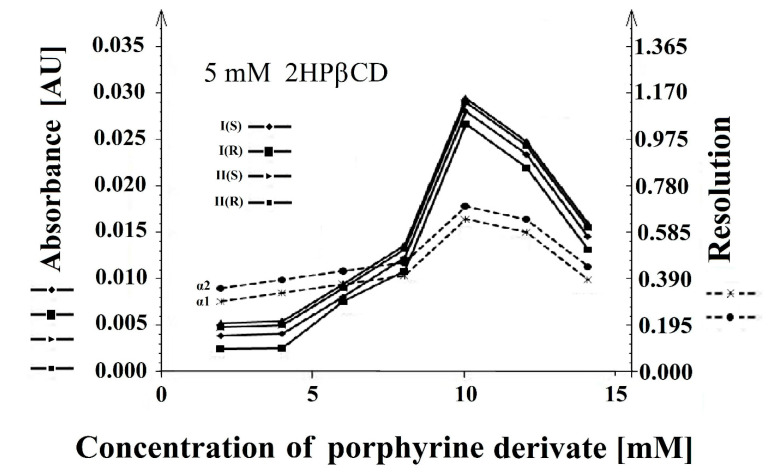
The influence of the concentration of 5,10,15,20-tetrakis (4-hydroxyphenyl) porphyrin at a constant concentration of 2-hydroxypropyl-β-cyclodextrin (2HPβCD) (5 mM) on the resolution (α1 and α2) and absorbance (AU) of the **I** (*S*), **I** (*R*) and **II** (*S*), **II** (*R*) enantiomers.

The resolution was tested by introducing the second selector-(5,10,15,20-tetrakis (4-hydroxyphenyl) porphyrin) into the system. Maintaining the concentration of 2-hydroxypropyl-β-cyclodextrin in the tested system at 5 mM, the concentration of 5,10,15,20-tetrakis (4-hydroxyphenyl) porphyrin was gradually increased while examining the resolution of the enantiomers **I** (*S*), **I** (*R*), **II** (*S*), and **II** (*R*). The best resolution and absorbance were achieved for the system containing 2-hydroxypropyl-β-cyclodextrin at a concentration of 5 mM and 5,10,15,20-tetrakis (4-hydroxyphenyl) porphyrin at a concentration of 10 mM (Figure 4).

The average detection times for **I** (*S*), **I** (*R*), **II** (*S*), and **II** (*R*) were 3.60, 4.08, 5.88, and 6.61 min, respectively (Figure 3B).

**Figure 5:**
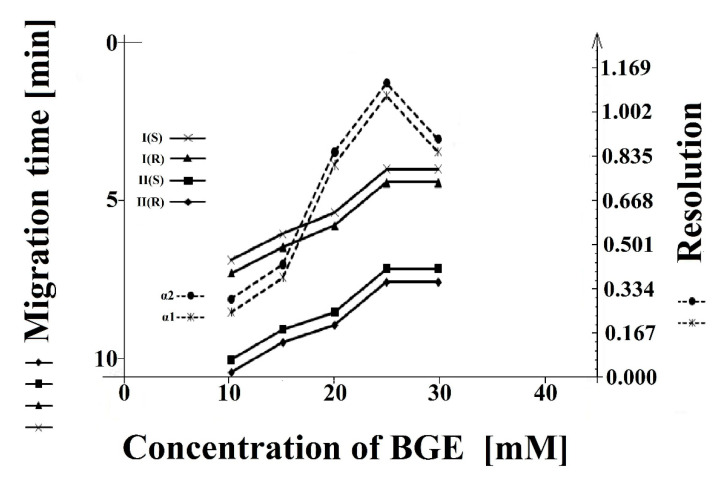
The influence of the concentration of background electrolyte buffer on the resolution (α1 and α2) and migration time of the **I** (*S*), **I** (*R*), and **II** (*S*), **II** (*R*) enantiomers.

**Figure 6:**
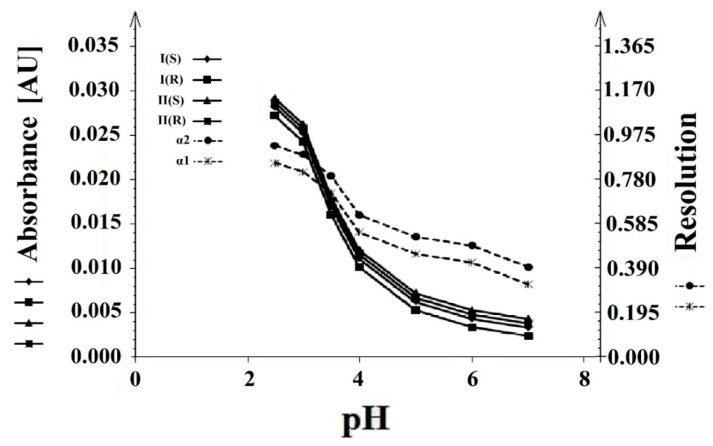
The influence of the pH buffer on the resolution (α1 and α2) and absorbance (AU) of the **I** (*S*), **I** (*R*), and **II** (*S*), **II** (*R*) enantiomers.

**Figure 7:**
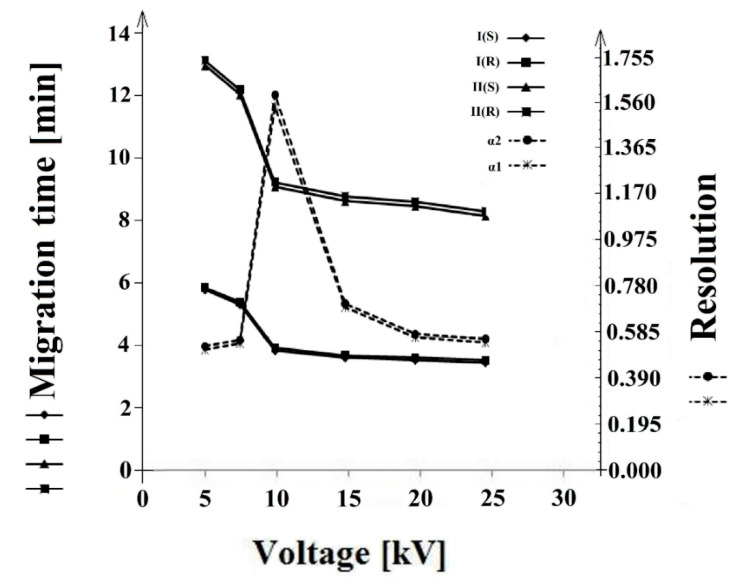
The influence of the voltage (kV) on the resolution (α1 and α2) and migration time of the **I** (*S*), **I** (*R*), and **II** (*S*), **II** (*R*) enantiomers.

**Figure 8:**
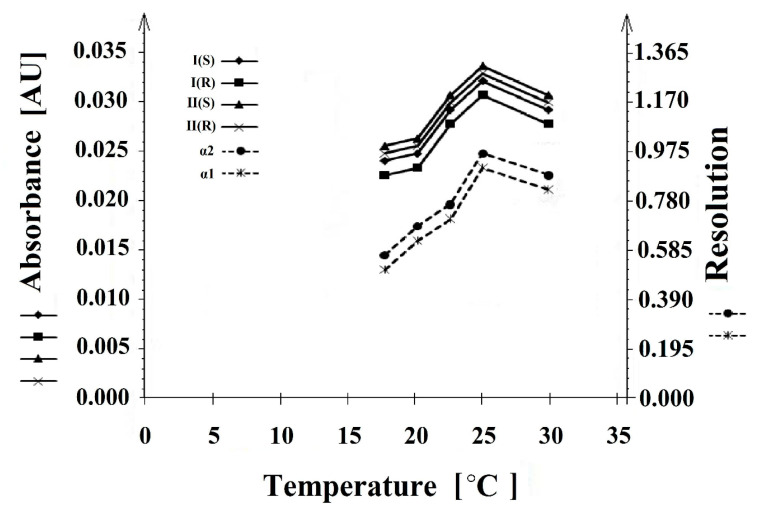
The influence of the temperature (T) on the resolution (α1 and α2) and migration time of the **I** (*S*), **I** (*R*), and **II** (*S*), **II** (*R*) enantiomers.

The combination of CDs with 5,10,15,20-tetrakis (4-hydroxyphenyl) porphyrin used as the second selector significantly improved the separation conditions such as time, resolution, and sensitivity for both the enantiomers of the compounds (**I**) and (**II**) (Figure 3B). The resolution presented in the Figure 4, Figure 5, Figure 6, Figure 7 and Figure 8 was calculated based on the migration time ratios for **I** (*S*), **I** (*R*), and **II** (*S*), **II** (*R*) according to the equations: equations:

(1) $$\alpha 1=\frac{tmI\left(R\right)}{tmI\left(S\right)}\;\mathrm{and}\;\alpha 2=\frac{tmII\left(R\right)}{tmII\left(S\right)}$$

where: α-resolution; tm **I** (*R*)-migration time of compound **I** (*R*) [min]; tm **I** (*S*)-migration time of compound **I** (*S*) [min]; tm **II** (*R*)-migration time of compound **II** (*R*) [min]; tm **II** (*S*)-migration time of compound **II** (*S*) [min]

The influence of BGE concentration on the resolution and migration time was investigated at 10, 15, 20, 25, and 30 mM. As the concentration of phosphate buffer increased, the resolution increased, the peak shapes sharpened, and the migration time of the determined compounds **I** (*S*), **I** (*R*), **II** (*S*), and **II** (*R*) decreased. The best results were observed at the concentration of 25 mM (Figure 5).

Considering the resolution, peak shape, and migration times of (**I**) and (**II**) compounds, the final BGE conditions were determined as follows: concentration: 25 mM and pH: 2.5.

The effect of pH on the separation process was analyzed at pH values from 7.0 to 2.5. A decrease in pH led to an increase in the resolution between (**I**) and (**II**) enantiomers. At pH 7.0, no resolution was found between the tested compounds. At pH 3.5, the resolution was insufficient between derivatives **I** (*S*), **I** (*R*), and **II** (*S*), **II** (*R*). The best resolution and absorbance of these compounds were observed with phosphate buffer at a concentration of 25 mM and pH 2.5 (Figure 6).

The influence of voltage on the separation and migration time was assessed at 5, 7, 10, 15, 20, and 25 kV. An increase in voltage to 10 kV increased the resolution. After 10 kV was exceeded, the resolution decreased, while the migration time decreased with the increasing voltage (Figure 7).

The influence of the temperature of the separation process was investigated at 18, 20, 22, 25, and 30°C. An increase in the temperature caused an increase in absorbance and resolution. The best results were achieved at 25°C (Figure 8).

Because the best separation of **I** (*S*), **I** (*R*), **II** (*S*), and **II** (*R*) was achieved using 2-hydroxypropyl-β-cyclodextrin and 5,10,15,20-tetrakis (4-hydroxyphenyl) porphyrin system at concentrations of 5 and 10 mM, respectively, in 25 mM phosphate buffer at a pH of 2.5, separation voltage of 10 kV, and temperature of 25 °C, this method was used for the preliminary studies of the enantiomers **I** (*S*), **I** (*R*), and **II** (*S*) and **II** (*R*) of the newly patented aminoalkanol derivatives in the blood serum (Figure 3D).

### 3.2. Method Development

CE enables us to selectively monitor **I** (*S*), **I** (*R*), and **II** (*S*) and **II** (*R*) and to eliminate interference with the endogenous components that can be co-eluted from serum samples. The enantiomers **I** (*S*), **I** (*R*), and **II** (*S*), **II** (*R*) were separated using 2-hydroxypropyl-β-cyclodextrin and 5,10,15,20-tetrakis (4-hydroxyphenyl) porphyrin system at the concentration of 5 and 10 mM, respectively. For developing this method as well as determining the validation parameters, the buffer pH, separating BGE concentration, chiral selector concentration, concentration and composition of extraction phase, wavelength, voltage, and temperature were optimized. During extraction, 500 μL of the serum solution was added in a glass tube, followed by which 100 μL of **I** (*S*) and **I** (*R*) and 100 μL of **II** (*S*) and **II** (*R*) were added, and the solutions were mixed together. The mixture was set aside for 5 min, and then, 5 mL of extraction solvent (*n*-hexane–ethyl acetate, 90:10, % *v*/*v*) was added. The mixture was shaken vigorously (vortexed) for 4 min. Subsequently, the mixture was centrifuged for 4 min at 1000× g. After centrifugation, the separated organic layer was removed and added to another glass tube, and was placed in a heating mantle, at a temperature of 37 °C. Then, it was subjected to evaporation at 37 °C under a nitrogen stream. The obtained residue was mixed with deionized water at 37 °C. Finally, the dissolved sample was moved to a capillary vial and then injected on the capillary. In this study, phosphate buffer containing 2-hydroxypropyl-β-cyclodextrin and 5,10,15,20-tetrakis (4-hydroxyphenyl) porphyrin system at a concentration of 5 and 10 mM, respectively, was chosen as a BGE because of its good separation performance. Considering the analysis time and resolution comprehensively, +10 kV was chosen as the separation voltage. Based on the above study, CE was applied for separation, with the separation conditions set as follows: BGE: 25 mM phosphate buffer at pH 2.5 containing 2-hydroxypropyl-β-cyclodextrin and 5,10,15,20-tetrakis (4-hydroxyphenyl) porphyrin system at 5 and 10 mM, respectively; voltage: +10 kV; detection temperature: 25 °C; and detection wavelength: 200 nm. The measurements were performed with a fused-silica capillary (effective length: 20 cm; diameter: 50 μm). The samples to be tested were hydrodynamically injected on the capillary [47]. Under these separation conditions, compounds (**I**) and (**II**) were eluted and separated in less than 7 min. The peaks were identified by injecting individual analytes based on migration time matching.

The preliminary analysis of the studied drugs exhibiting biological activity is described elsewhere [46,47]. The extraction procedure developed in this study required using an extraction solvent made of *n*-hexane and ethyl acetate, in 90:10 (% *v*/*v*) proportions. The optimum time of sample shaking was determined as 4 min. Appropriate organic layer separation was performed by centrifuging at 1000× g for 5 min. The separated organic layer was subjected to evaporation under a nitrogen stream at 37 °C. Deionized water was added to dissolve the samples, after which the samples were injected on the capillary. The developed extraction procedure is a simple, time-saving, and single-step process.

### 3.3. Method Validation

The linearity, precision, specificity, accuracy, carry-over extraction recoveries, and limit of detection (LOD) as well as limit of quantification (LOQ) of the developed method were validated by following the guidelines of the International Council on Harmonization (ICH) [48] and the previously presented procedure [47].

The first step before the validation process involved the screening of selectors, as described in Section 3.1. Optimization.

The specificity of the method was defined as its ability to differentiate and quantify an analyte of interest and an internal standard from the endogenous components in the matrix, or other components in the sample. The presence of potential endogenous interference was assessed by analyzing 10 serum samples obtained from the free-drug volunteers, which were fortified with the **I** (*S*), **I** (*R*), and **II** (*S*), **II** (*R*) compounds. The measurement results (Figure 3C) showed the absence of endogenous interferences for every analyte of interest. The LODs and LOQs were determined by measuring the **I** (*S*), **I** (*R*), and **II** (*S*), **II** (*R*) compounds in a series of decreasing concentrations in the serum samples. In line with the ICH guidelines, LODs were determined at the lowest concentrations of **I** (*S*), **I** (*R*), and **II** (*S*), **II** (*R*), with a signal-to-noise ratio of at least 3 for all the compounds. The LOQ values were determined by quantitative measurement of the lowest compound concentration with a stated and acceptable accuracy and precision (CV% <20). The LODs and LOQs were estimated by analyzing the fortified serum samples of 500 µL volume. All the calibration curves indicated an excellent linear regression (with an *R*^2^ of 0.9969–0.9998). The LOD values for **I** (*S*), **I** (*R*), **II** (*S*), and **II** (*R*) were estimated at 65.2, 65.6, 65.4, and 65.7 ng/mL, respectively, while the LOQ values for these compounds were 216.5, 217.8, 217.1, and 218.1 ng/mL, respectively (Table 1).

Recovery of the compounds **I** (*S*), **I** (*R*), **II** (*S*), and **II** (*R*) was assessed by comparing the mean relative peak area for the samples containing these compounds at three different levels of concentration (high, medium, and low) in relation to the true concentrations of the pure standards, with the mean relative peak area for those compounds after extraction from the matrix. The recoveries determined for the analytes from serum solution and water were satisfactory in a range of 94.6–99.9% (Table 2). The results presented in Table 2 show that the extraction method is suitable for measuring the drug levels in patients’ serum.

The precision of the proposed method in terms of repeatability was determined by conducting a replicate analysis (*n* = 6) of serum extracts that were spiked with 100 and 1000 ng/mL of each compound. The relative standard deviation for migration time and relative peak area was less than 3.0% (Table 3).

Stability tests were performed under high-quality control. The analytes were stable in a serum blank at room temperature for 6 h and under repeated freeze–thaw conditions (totally three cycles). The samples to be tested were kept at −25 °C to evaluate their stability in a short-term experiment. The findings presented in Table 4 confirm the stability of the analytes for around 30 days.

Carry-over was estimated by injecting blank samples after a mixture of analytes. The blank samples injected immediately after the samples containing 1000 ng/mL of each compound showed no evidence of carry-over.

### 3.4. Application of the CE Method for the Quantitation of **I** (S), **I** (R) and **II** (S), **II** (R) Enantiomers in Real Serum Samples

The developed CE method was employed in the serum samples to quantify the enantiomers **I** (*S*), **I** (*R*), **II** (*S*), and **II** (*R*) of potential anticancer drugs, which are the new derivatives (**I)** and (**II)** of 1,7-diethyl-8,9-diphenyl-4-azatricyclo[5.2.1.0^2,6^]dec-8-ene-3,5,10-trione. Serum taken from healthy volunteers was used as the matrix and spiked with enantiomer standards of compounds (**I**) and (**II**). The expected concentration of the particular compounds in patient serum samples was 45 µg/mL. The developed method enabled determining the **I** (*S*), **I** (*R*), **II** (*S*), and **II** (*R*) compounds at concentrations 216.5 ng/mL, 217.8 ng/mL, 217.1 ng/mL, and 218.1 ng/mL for **I** (*S*), **I** (*R*), **II** (*S*), and **II** (*R*) compounds, respectively. Furthermore, the expected concentration was much higher than the LOD of the developed method, which indicates that the method can be successfully applied for the determination of individual enantiomers of compounds (**I**) and (**II**) in serum.

## 4. Conclusions

This paper described the procedure that uses the dual chiral-achiral selector complex consisting of 2-hydroxypropyl-β-cyclodextrin and 5,10,15,20-tetrakis (4-hydroxyphenyl) porphyrin in CE for the concomitant detection of the four enantiomers of anticancer drugs, the newly patented **I** and **II** derivatives of 1,7-diethyl-8,9-diphenyl-4-azatricyclo[5.2.1.0^2,6^]dec-8-ene-3,5,10-trione. The present study showed that the developed method allows separating a mixture of molecules **I** (*S*), **I** (*R*), and **II** (*S*), **II** (*R*), and is stereospecific, accurate and precise, and suitable for studying the pharmacokinetics, bioavailability, and optical purity of these enantiomers. In addition, the method enables a good separation of the enantiomers **I** (*S*), **I** (*R*), and **II** (*S*), **II** (*R*), in a very short time, with a lower amount of the injected sample. The single-step method of extraction offers an excellent recovery at a range of 94.6–99.9%**.** Furthermore, the extraction procedure is easy to perform and time saving, and hence can be used in clinical trial studies. The LOQs were 216.5 ng/mL for compound **I** (*S*), 217.8 ng/mL for compound **I** (*R*), 217.1 ng/mL for compound **II** (*S*), and 218.1 ng/mL for compound **II** (*R*). The run time was found to be only 8 min for each sample. Among the three CDs tested as chiral selectors, only 2-hydroxypropyl-β-cyclodextrin allowed for the separation of **I** (*S*), **I** (*R*), and **II** (*S*), **II** (*R*) enantiomers. The introduction of 5,10,15,20-tetrakis (4-hydroxyphenyl) porphyrin as the second selector and creation of the dual 2-hydroxypropyl-β-cyclodextrin and 5,10,15,20-tetrakis (4-hydroxyphenyl) porphyrin chiral-achiral selector system led to a significant improvement in the separation process and sensitivity of the method, and thus allowed achieving very good LODs and determining the enantiomers of compounds (**I**) and (**II**).

The porphyrin derivative used in this study most likely creates additional connections with the previously formed complexes of individual enantiomers of compounds I and II with 2-hydroxypropyl-β-cyclodextrin, creating a kind of additional molecular sieve increasing the molecular weight of the complexes, their absorbance, and resolution.

In addition, the developed CE method was applied in the pilot studies of **I** (*S*), **I** (*R*), **II** (*S*), and **II** (*R*) enantiomers in the blood serum and yielded great results.

## Figures and Tables

**Table 1:** Regression equation, limits of detection, and quantification for compounds I(*S*), I(*R*), and II(*S*), II(*R*) (*n* = 6).

Compounds	Linearity Range	R^2^	(RSD) [%]	LOD	LOQ	Regression Equation	(± SD)	
	ng/mL	Mean		ng/mL	ng/mL	Mean	Slope	Intercept
**I**(*S*)	200–10000	0.9998	1.62	65.2	216.5	y = 51.80x − 1425	±0.11	±5.41
**I**(*R*)	200–10000	0.9979	2.17	65.6	217.8	y = 51.81x − 1477	±0.12	±6.13
**II**(*S*)	200–10000	0.9998	2.03	65.4	217.1	y = 51.80x − 1415	±0.11	±5.50
**II**(*R*)	200–10000	0.9969	2.23	65.7	218.1	y = 49.68x − 994	±0.14	±7.04

RSD–Relative Standard Deviation; LOD–Limit Of Detection; LOQ–Limit Of Quantification.

**Table 2:** Recovery data of compounds **I** and **II** from standard solutions **(SL)** (*n* = 6) and serum **(*S*)** (*n* = 6).

Compounds	Added Amount	Observed Amount	%Recovery	%RSD	Compounds	Added Amount	Observed Amount	%Recovery	%RDS
		Mean ± SD					Mean ± SD		
**(SL)**	ng/mL	ng/mL			**(S)**	ng/mL	ng/mL		
**I** (*S*)	100	98.9 ± 3.5	98.9	3.5	**I** (*S*)	100	95.8 ± 5.3	95.8	5.5
	500	495.2 ± 2.7	99.4	0.5		500	496.7 ± 3.4	99.3	0.7
	1000	998.9 ± 1.2	99.9	0.1		1000	997.9 ± 2.2	99.8	0.2
**I** (*R)*	100	97.8 ± 3.9	97.8	4.0	**I** (*R)*	100	94.9 ± 5.9	94.9	6.2
	500	496.9 ± 3.1	99.4	0.6		500	497.1 ± 2.9	99.4	0.6
	1000	997.7 ± 1.6	99.8	0.2		1000	996.8 ± 3.3	99.7	0.3
**II** (*S)*	100	97.4 ± 3.7	97.4	3.8	**II** (*S*)	100	95.3 ± 4.9	95.3	5.1
	500	495.0 ± 2.9	99.0	0.6		500	498.6 ± 1.4	99.7	0.3
	1000	998.0 ± 1.5	99.8	0.2		1000	998.7 ± 1.3	99.9	0.1
**II** (*R)*	100	97.6 ± 3.4	97.6	3.5	**II** (*R)*	100	94.6 ± 5.5	94.6	5.8
	500	496.0 ± 2.9	99.2	0.6		500	496.5 ± 3.6	99.3	0.7
	1000	997.0 ± 2.2	99.7	0.2		1000	997.1 ± 3.0	99.7	0.3

**Table 3:** Intra-day and inter-day precision of compounds **I (*S*), I (*R*)** and **II (*S*), II (*R*)** analytes.

Compounds	Concentration	Intra-Day Precision (*n* = 6, Mean)	Inter-Dday Precision						
	ng/mL	Day-1	Day-2	Day-3	(*n* = 18, mean)				
		*t*_m_/%RSD	*P*_A_/%RSD	*t*_m_/%RSD	*P*_A_/%RSD	*t*_m_/%RSD	*P*_A_/%RSD	*t*_m_/%RSD	*P*_A_/%RSD
I (*S*)	100	3.72/0.5	1116/1.0	3.79/0.5	1138/1.0	3.75/0.5	1093/1.0	3.75/1.1	1116/2.0
I (*R*)	100	4.37/0.4	1150/1.0	4.39/0.7	1172/1.0	4.42/0.7	1138/1.0	4.39/0.7	1153/1.5
II (*S*)	100	6.08/0.5	1003/ 0.9	6.12/0.5	1116/1.0	6.13/0.5	1071/1.0	6.11/0.5	1063/2.9
II (*R*)	100	7.23/0.6	1195/1.1	7.28/0.5	1138/1.0	7.25/0.6	1161/1.0	7.25/0.4	1165/2.5
I (*S*)	1000	3.61/0.3	12389/0.4	3.67/0.3	14247/0.5	3.65/0.3	13938/0.4	3.64/0.8	13525/0.5
I (*R*)	1000	4.16/0.2	13938/0.4	4.25/0.2	14557/0.5	4.23/0.2	13628/0,4	4.21/1.2	14041/0.5
II (*S*)	1000	5.90/0.2	9911/0.3	5.95/0.2	11770/0.4	5.98/0.3	11150/0.4	5.94/0.7	10944/0.4
II (*R*)	1000	7.00/0.3	13628/0.4	7.04/0.3	13938/0.4	7.08/0.3	13318/0.4	7.04/0.6	13628/0.4

*t*_m_—migration time; *P*_A_—peak area.

**Table 4:** Stability studies for compounds **I** (*S*), **I** (*R*), and **II** (*S*), **II** (*R*).

Spiked	Bench Top ^a^	Freeze and Thaw ^b^	Short Term ^c^				
Concentration							
(ng/mL)	_________________	________________	_________________				
	Mean ± SD	Mean ± SD	Mean ± SD				
	Obtained	%RSD	Obtained	%RSD	Obtained	%RSD	
	Concentration		Concentration		Concentration		
	(ng/mL) ^d^	(ng/mL) ^d^	(ng/mL) ^d^				
**I** (*S*)	100	98.8 ± 3.2	3.2	91.2 ± 4.9	5.4	100.9 ± 4.9	4.9
	1000	999.0 ± 1.3	0.1	994.1 ± 2.8	0.3	1001.2 ± 2.8	0.3
**I** (*R*)	100	97.3 ± 3.9	4.0	90.9 ± 5.0	5.5	100.1 ± 4.2	4.2
	1000	998.6 ± 2.6	0.3	990.5 ± 3.3	0.3	1003.7 ± 3.3	0.3
**II** (*S*)	100	99.8 ± 1.5	1.5	96.2 ± 3.1	3.2	101.4 ± 3.9	3.8
	1000	1000.0 ± 1.3	0.1	996.1 ± 3.8	0.4	1002.2 ± 2.9	0.3
**II** (*R*)	100	97.9 ± 2.9	3.0	94.3 ± 4.1	4.3	104.2 ± 3.7	3.6
	1000	998.4 ± 2.6	0.3	991.3 ± 3.1	0.3	1002.7 ± 2.1	0.2

^a^ After 6 h at room temperature. ^b^ After three freeze and thaw cycles at −20 ◦C. ^c^ At −25 °C for 30 days. ^d^ Values are mean ± SD for five replicates.

## Data Availability

Not applicable.
